# Trends in post-discharge major adverse cardiovascular events following myocardial infarction: Insights from the veterans healthcare administration

**DOI:** 10.1016/j.ajpc.2026.101626

**Published:** 2026-04-09

**Authors:** Alexander T Sandhu, Adam Furst, Fatima Rodriguez, Neil Kalwani, David J. Maron, Shriram Nallamshetty, Natasha Din, Michael Fan, Kat Khachatourian, Jeffrey R. Skaar, Ivy Tonnu-Mihara

**Affiliations:** a Greater Los Angeles Veterans Affairs Healthcare System, Los Angeles, CA, United States; b University of California Los Angeles, Los Angeles, CA, United States; c Palo Alto Veterans Affairs Healthcare System, Palo Alto, CA, United States; d Division of Cardiology, Stanford University School of Medicine, Stanford, CA, United States; e Stanford Prevention Research Center, Stanford University School of Medicine, Stanford, CA, United States; f Medical Affairs, Novo Nordisk Inc., Plainsboro, NJ, United States; g Clinical Data Science and Evidence, Novo Nordisk Inc., Plainsboro, NJ, United States

**Keywords:** Myocardial infarction, Heart failure, Epidemiology

## 1 Background

Contemporary treatment of myocardial infarction (MI) has led to improved in-hospital survival [1]. However, patients who survive an MI hospitalization remain at increased risk for post-discharge adverse events. Understanding temporal changes in post-discharge outcomes is critical to efforts to improve long-term outcomes. We used the Veterans Health Administration (VHA) to evaluate long-term trends in post-discharge outcomes for patients with a non-fatal MI from 2008–2022.

## 2 Methods

We used data from VHA electronic health records (EHR), Medicare claims, and VHA community care to identify Veterans hospitalized for a non-fatal MI from 2008–2022. MI hospitalization was defined using principal diagnosis (based on an International Classification of Disease-9/10 [ICD] codes corresponding to MI) [2,3]. For Veterans with multiple MI hospitalizations, we randomly selected one as the index event to create a representative cross-sectional cohort.

We identified Veteran characteristics in the two years preceding the index MI. We used the most recent vital signs and laboratory values. The index MI was classified as an ST-segment elevation MI (STEMI) or non–ST-segment elevation MI (NSTEMI) based on the diagnosis code. Inpatient coronary revascularization (percutaneous coronary intervention [PCI] and coronary artery bypass grafting [CABG]) were identified based on procedural codes. NSTEMI were also stratified between those with and without revascularization to account for the increasing recognition of Type II MI over time. For missing vital signs and laboratory values, we used multiple imputation with chained equations with 30 imputed datasets.

The primary outcome was the composite major adverse cardiovascular endpoint of all-cause mortality, recurrent MI, ischemic stroke, or HF hospitalization at 1 year post-MI. Recurrent MI, ischemic stroke and HF hospitalization were identified based on principal diagnoses. Each of these components was also evaluated individually. We also evaluated cardiovascular death defined by a cardiovascular ICD code (I.00-I.99) for the underlying cause of death.

Trends in outcomes were evaluated using the Cuzick rank test to detect ordered differences across three time periods: 2008–2012, 2013–2017, and 2018–2022. We used multivariable logistic regression to estimate the annual relative change in the odds of each outcome, adjusting for age, sex, comorbidities, frailty, preadmission heart rate, systolic blood pressure, LDL cholesterol, estimated glomerular filtration rate, hemoglobin A1c, and MI classification (STEMI or NSTEMI). Continuous variables were modeled as restricted cubic splines. The year of the index MI was modeled as a continuous variable. Imputed datasets were combined via Rubin’s rule. We repeated the analyses stratified by (1) STEMI vs. NSTEMI with revascularization vs. NSTEMI without revascularization and (2) with revascularization vs. without revascularization.

Finally, we performed a landmark analysis comparing the risk of MACE between 12–24 months after index MI between Veterans with versus without HF hospitalization in the first 12 months post-MI. This analysis excluded Veterans who died within 12 months post-MI. We used a two-sided p-value threshold of 0.05 to identify statistical significance.

## 3 Results

We identified 444,659 Veterans hospitalized for a non-fatal MI. The median age was 73 years (interquartile range [IQR]: 66–80) and 1.8 % were women. From 2008–2012, 143,903 Veterans had a median age of 74 (IQR: 65–82) years, with 15 % with chronic kidney disease and 32 % with diabetes mellitus. From 2013–2017, the 153,142 Veterans had a median age of 71 (IQR: 66–80), with 22 % with chronic kidney disease and 38 % with diabetes mellitus. From 2018–2022, 147,614 Veterans had median age of 73 (IQR: 67–79), with 28 % with chronic kidney disease and 46 % with diabetes mellitus. STEMI hospitalizations decreased from 26.1 % of MI in 2008–2012, to 23.1 % in 2013–2017, and remained at 23.1 % in 2018–2022 (*p* < 0.01 for trend). Over the same intervals, inpatient revascularization decreased from 49.6 % to 48.1 % to 36.9 % (*p* < 0.01). Among NSTEMI hospitalizations, the rate of inpatient revascularization decreased from 41.1 % to 40.9 % to 32.6 % (*p* < 0.01).

**Fig. 1:**
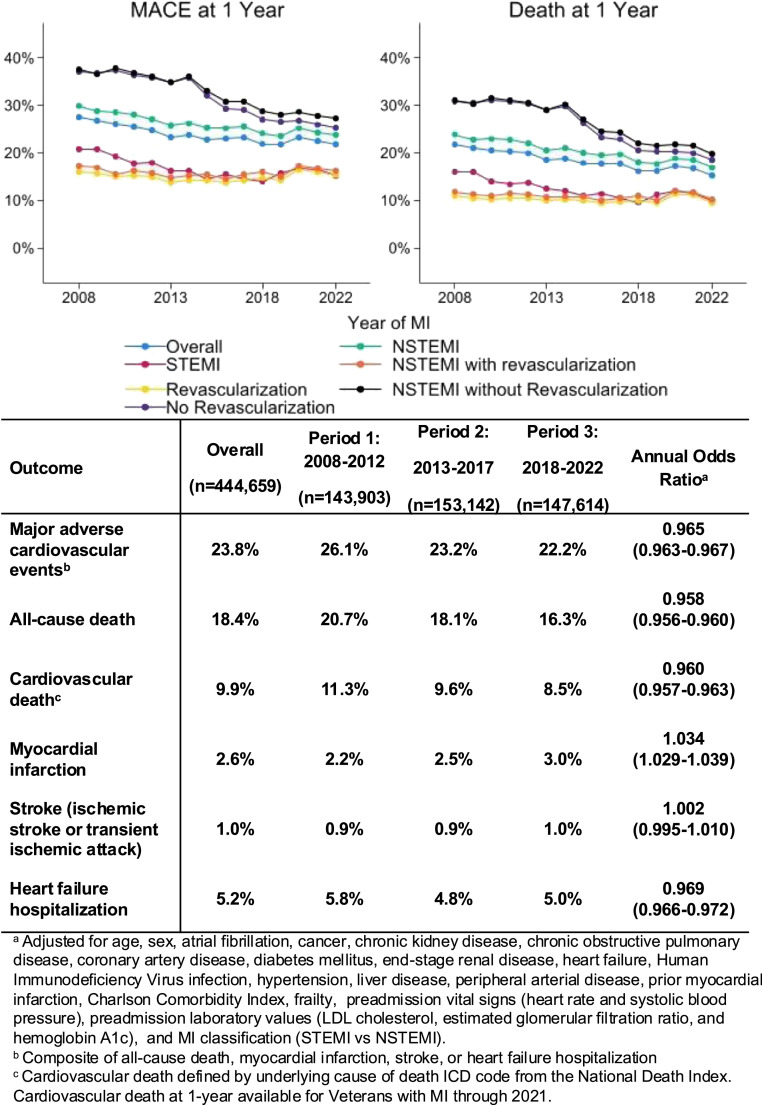
Trends in clinical outcomes in the one year following MI hospitalization.

Within 1 year following hospital discharge for MI, the unadjusted risk of MACE declined significantly across the three periods, from 26.1 % in 2008–2012 to 23.2 % in 2013–2017 to 22.2 % in 2018–2022 (*p* < 0.01) (Fig. 1). After adjustment, there was a 3.5 % annual relative decrease in the odds of experiencing MACE (odds ratio [OR]: 0.965 per year; 95 % confidence interval [CI]: 0.963–0.967). We found similar patterns between Veterans with STEMI (OR: 0.951; 95 % CI: 0.947–0.956), NSTEMI with revascularization (OR: 0.973; 95 % CI: 0.969–0.977), and NSTEMI without revascularization (OR: 0.965; 95 % CI: 0.962–0.967). There was also an annual decrease in the odds of MACE among MI with revascularization (OR: 0.967; 95 % CI: 0.964–0.971) and without revascularization (OR: 0.959; 95 % CI: 0.956–0.961).

The 1-year risk of death post-MI hospitalization also substantially decreased over time, from 20.7 % to 18.1 % to 16.3 % (*p* < 0.01). After adjustment, we observed a 4.3 % annual relative decrease in the odds of 1-year mortality (OR: 0.958 per year; 95 % CI: 0.956–0.960).

There were 23,048 Veterans (5.2 %) with a HF hospitalization in the first year post-MI. Among the 348,345 Veterans who survived to 1 year post-MI, the mortality rate over the subsequent year was 28.6 % for those with a HF hospitalization compared with 9.1 % among those without a HF hospitalization in the first year post-MI (*p* < 0.001 between groups). We observed 1.5 % annual relative decrease in the odds of death after the HF hospitalization (OR 0.985; 95 % CI: 0.978–0.993).

## 4 Discussion

Among 444,000 Veterans hospitalized between 2008–2022, post-discharge outcomes after MI improved. These reductions in adverse outcomes may reflect improved acute care management and post-discharge medical therapy. However, over 20 % of patients still experienced MACE within 1 year, illustrating that MI remains a significant risk factor for poor long-term outcomes. These results extend prior analyses demonstrating improvement in MI outcomes among Medicare patients [1].

Temporal improvements were noted for the composite MACE outcome, all-cause death, and heart failure hospitalization, but not for ischemic stroke or recurrent MI. The discrepancy with recurrent MI may relate to changes in MI identification with increased sensitivity of troponin assays over time and increased identification of Type II MI. In a recent analysis, the use of high-sensitivity troponin assays increased from 3 % in 2019 to 33 % by 2021 [4]. Temporal change in the NSTEMI cohort may have influenced the observed improvements in outcomes. However, we found similar temporal improvements in outcomes among patients with STEMI and patients with NSTEMI with revascularization. These subgroups are less likely to be influenced by the increased identification of Type II MI.

Veterans that suffer subsequent HF hospitalizations post-MI are at particularly elevated risk. This is consistent with prior findings [5]. This illustrates the importance of strategies for HF prevention following MI hospitalization. Identifying patients who are most likely to develop HF and intervening proactively may further reduce post-discharge morbidity.

There are multiple important limitations. First, MI identification relied on ICD codes. This is important given the evolution of troponin assays and changing diagnostic practices over time. Second, we did not examine trends in evidence-based therapies during the hospitalization and post-discharge. Future research should aim to better understand the change in therapies that are associated with improvement in outcomes. Finally, the VHA population is predominantly male and older, which may limit generalizability to more diverse populations.

In a large cohort of Veterans who survived hospitalization for MI from 2008–2022, the 1-year risk of MACE and death decreased significantly. Nonetheless, the risk of adverse outcomes remains high, especially among those who develop HF. Additional strategies to reduce morbidity following MI are needed.

## CRediT authorship contribution statement

**Alexander T Sandhu:** Writing – original draft, Supervision, Methodology, Funding acquisition, Formal analysis, Data curation, Conceptualization. **Adam Furst:** Writing – review & editing, Methodology, Formal analysis. **Fatima Rodriguez:** Writing – review & editing, Investigation. **Neil Kalwani:** Writing – review & editing, Investigation. **David J. Maron:** Writing – review & editing, Investigation. **Shriram Nallamshetty:** Writing – review & editing, Investigation. **Natasha Din:** Writing – review & editing, Investigation. **Michael Fan:** Writing – review & editing, Methodology, Investigation. **Kat Khachatourian:** Writing – review & editing, Methodology, Investigation. **Jeffrey R. Skaar:** Writing – review & editing, Methodology, Investigation. **Ivy Tonnu-Mihara:** Writing – review & editing, Supervision, Methodology, Investigation, Conceptualization.

## Declaration of competing interest

The authors declare the following financial interests/personal relationships which may be considered as potential competing interests: Alexander Sandhu reports financial support was provided by Novo Nordisk Inc. Alexander Sandhu reports a relationship with 10.13039/100002429Amgen that includes: funding grants. Alexander Sandhu reports a relationship with Astra Zeneca that includes: funding grants. Alexander Sandhu reports a relationship with Bayer AG that includes: funding grants. Alexander Sandhu reports a relationship with 10.13039/100008272Novartis Pharmaceuticals Corporation that includes: funding grants. Alexander Sandhu reports a relationship with Reprieve Cardiovascular that includes: consulting or advisory. Alexander Sandhu reports a relationship with Holosis that includes: consulting or advisory. Alexander Sandhu reports a relationship with Cleerly that includes: consulting or advisory. Fatima Rodriguez reports a relationship with Carta Healthcare Inc that includes: equity or stocks. Fatima Rodriguez reports a relationship with HealthPals that includes: consulting or advisory and equity or stocks. Fatima Rodriguez reports a relationship with Novartis Pharmaceuticals Corporation that includes: consulting or advisory. Fatima Rodriguez reports a relationship with Novo Nordisk Inc that includes: consulting or advisory. Fatima Rodriguez reports a relationship with Esperion Therapeutics Inc that includes: consulting or advisory. Fatima Rodriguez reports a relationship with Movano Health that includes: consulting or advisory. Fatima Rodriguez reports a relationship with Kentho Health that includes: consulting or advisory. Fatima Rodriguez reports a relationship with Inclusive Health that includes: consulting or advisory. Fatima Rodriguez reports a relationship with Edwards Lifesciences Corporation that includes: consulting or advisory. Fatima Rodriguez reports a relationship with Cleerly Inc that includes: consulting or advisory. Fatima Rodriguez reports a relationship with Arrowhead Pharmaceuticals Inc that includes: consulting or advisory. Fatima Rodriguez reports a relationship with iRhythm Technologies Inc that includes: consulting or advisory. Fatima Rodriguez reports a relationship with HeartFlow Inc that includes: consulting or advisory. David Maron reports a relationship with Ablative Solutions Inc that includes: equity or stocks. David Maron reports a relationship with Preemptive AI that includes: equity or stocks. David Maron reports a relationship with JVMP Labs that includes: equity or stocks. David Maron reports a relationship with Cleerly Inc that includes: funding grants. David Maron reports a relationship with Omada Health that includes: funding grants. David Maron reports a relationship with HeartFlow Inc that includes: consulting or advisory. David Maron reports a relationship with Inno Med that includes: consulting or advisory. David Maron reports a relationship with Johnson and Johnson that includes: consulting or advisory. David Maron reports a relationship with Regeneron Pharmaceuticals Inc that includes: consulting or advisory. David Maron reports a relationship with SCILEX Pharmaceuticals that includes: consulting or advisory. David Maron reports a relationship with New Amsterdam School that includes: consulting or advisory. David Maron reports a relationship with Bayer AG that includes: consulting or advisory. Michael Fan reports a relationship with Novo Nordisk Inc that includes: employment. Kat Khachatourian reports a relationship with Novo Nordisk Inc that includes: employment and equity or stocks. Jeffrey R. Skaar reports a relationship with Novo Nordisk Inc that includes: employment and equity or stocks. Jeffrey R. Skaar reports a relationship with Trinity Life Science that includes: equity or stocks. Ivy Tonnu-Mihara reports a relationship with Novo Nordisk Inc that includes: employment. If there are other authors, they declare that they have no known competing financial interests or personal relationships that could have appeared to influence the work reported in this paper.
